# Bmi1 Is Down-Regulated in the Aging Brain and Displays Antioxidant and Protective Activities in Neurons

**DOI:** 10.1371/journal.pone.0031870

**Published:** 2012-02-23

**Authors:** Mohamed Abdouh, Wassim Chatoo, Jida El Hajjar, Jocelyn David, José Ferreira, Gilbert Bernier

**Affiliations:** 1 Developmental Biology Laboratory, Hôpital Maisonneuve-Rosemont, Montréal, Canada; 2 Department of Pathology, Hôpital Maisonneuve-Rosemont, Montréal, Canada; 3 Department of Ophthalmology, Université de Montréal, Montréal, Canada; Case Western Reserve University, United States of America

## Abstract

Aging increases the risk to develop several neurodegenerative diseases, although the underlying mechanisms are poorly understood. Inactivation of the Polycomb group gene Bmi1 in mice results in growth retardation, cerebellar degeneration, and development of a premature aging-like phenotype. This progeroid phenotype is characterized by formation of lens cataracts, apoptosis of cortical neurons, and increase of reactive oxygen species (ROS) concentrations, owing to p53-mediated repression of antioxidant response (AOR) genes. Herein we report that Bmi1 expression progressively declines in the neurons of aging mouse and human brains. In old brains, p53 accumulates at the promoter of AOR genes, correlating with a repressed chromatin state, down-regulation of AOR genes, and increased oxidative damages to lipids and DNA. Comparative gene expression analysis further revealed that aging brains display an up-regulation of the senescence-associated genes IL-6, p19^Arf^ and p16^Ink4a^, along with the pro-apoptotic gene Noxa, as seen in *Bmi1*-null mice. Increasing Bmi1 expression in cortical neurons conferred robust protection against DNA damage-induced cell death or mitochondrial poisoning, and resulted in suppression of ROS through activation of AOR genes. These observations unveil that *Bmi1* genetic deficiency recapitulates aspects of physiological brain aging and that Bmi1 over-expression is a potential therapeutic modality against neurodegeneration.

## Introduction

Aging is the prime risk factor for the development of several neurodegenerative diseases. Although the reason for this is unknown, it is possible that specific neuronal genes having protective roles in the aging brain are down-regulated due to increased mutational rate and/or defective DNA repair at their promoter [1]. Moreover, long lived neuronal cells are more likely to accumulate mutations in their genomic DNA than most other cell types with age, leading to impaired cellular functions [2], [3]. This phenomenon may be explain in part due to the inability of post-mitotic cells to replicate their DNA, a process that is tightly coupled to DNA damage checkpoint and DNA repair [4], [5], [6]. Moreover, neurons possess a high metabolic activity and consume large amount of O_2_, and thus are exposed to higher levels of oxidative stress compared to other tissues [3], [6].

Mitochondria are not only cellular organelles required for aerobic respiration but are also the main source of intracellular reactive oxygen species (ROS), which are thought to be causal for most oxidative damage accumulation with age [3], [7], [8]. The balance between ROS and antioxidant molecules is critical to determine the rate of oxidative damage accumulation, and thus possibly cellular and organism lifespan [9]. The free radical theory of aging is partially supported by gain- and loss-of-function experiments showing that antioxidant-encoding genes such as catalase, Cu/Zn superoxide dismutase (Sod1) and Mn superoxide dismutase (Sod2) are critical determinants of lifespan [10], [11], [12], [13]. Thus, reduced antioxidant response (AOR) genes expression in the aging brain could displace the oxidative balance and may instigate accelerated aging and age-related neurodegenerative diseases.

Polycomb group proteins form large multimeric complexes that silence specific target genes by modifying chromatin organization [14]. The Polycomb group protein Bmi1 is a component of the Polycomb Repressive Complex 1 (PRC1), which promotes chromatin compaction and gene repression through its histone H2A E3-mono-ubiquitin ligase activity [15], [16], [17]. *Bmi1^−/−^* mice present axial skeleton defects, reduced post-natal growth and lifespan, along with progressive cerebellar degeneration [18], [19]. Yet, we previously reported that *Bmi1^−/−^* mice develop a progeroid phenotype in the central nervous system characterized by lens cataracts, cortical neurons apoptosis, p53 activation and oxidative damage accumulation [20]. *Bmi1^−/−^* neurons are also hypersensitive to mitochondrial toxins, to DNA-damaging agents and to intracellular β-amyloid peptides, and display elevated ROS concentrations due to severely reduced AOR genes expression. Notably, most of Bmi1 functions on the oxidative metabolism of neurons can be explained by p53-mediated repression of AOR genes expression [20], [21].

In this study, we investigated the possible molecular links between physiological brain aging and *Bmi1* genetic deficiency, and analyzed Bmi1 neuroprotective potential. We show that in the aging brain, where Bmi1 expression declines in the neurons, there is an up-regulation of inflammatory and senescence markers, accumulation of p53 to AOR gene promoters, reduced expression of AOR genes and elevated oxidative damage to neurons, in a pattern that is similar to that of *Bmi1^−/−^* mice. Increasing Bmi1 expression in cortical neurons was highly neuroprotective against inhibition of the mitochondria respiratory chain or the DNA topoisomerase I, and resulted in activation of antioxidant defenses and in suppression of ROS levels. This work suggests that modulation of Bmi1 expression in the aging or disease-affected brain could provide robust neuroprotective activity.

## Materials and Methods

### Ethical statement

Animals were maintained in our facilities and used in accordance with and after approval by the Animal Care Committee of the Maisonneuve-Rosemont Hospital Research Center (Approval ID #2008-27 and #2009-42). Paraffin-embedded archival brains were obtained from the department of pathology of the Maisonneuve-Rosemont Hospital. Post-mortem human eyes were provided by the Eye's Bank of our institution and used with permission of Maisonneuve-Rosemont Hospital ethical committee.

### Adenovirus/plasmid constructs

Human BMI1 (−436 to +2175; GeneBank Accession # NM_005180) cDNA was cloned by PCR using human retina cDNA, and inserted into the DUAL-IRESGFP plasmid for adenovirus production (Vector Biolabs). BMI1 cDNA was also cloned into the CMV-GFP:EF1α lentiviral vector.

### Neuronal cultures

Embryonic day 18.5 cortical neurons were cultured as described previously [20]. Cortices were dissected in oxygenated HBSS. Following meninges removal, cortices were cut to ∼1 mm^3^ pieces, and incubated at 37°C for 15 min in 2 ml TrypleEx solution (Invitrogen). Afterwards, enzymatic solution was discarded, and cortex pieces dissociated in HBSS with a 1 ml tip (10 times up and down). After dissociation, cells were plated at 1.5×10^5^ cells/well on poly-L-lysine-coated 6-well plates or 8-well cultures slides (BD Biosciences). Cells were maintained in normal medium composed of Neurobasal-A Medium (Invitrogen), Glutamax-I (Gibco), gentamycin (50 µg/ml; Gibco), B27 supplement (Gibco), NGF (50 ng/ml; Invitrogen) and BDNF (0.5 ng/ml; Invitrogen). Neurons were nucleofected with plasmid DNA using the Mouse Neuron Nucleofector Kit according to manufacturer's instructions (Amaxa Biosystems).

### Chromatin immunoprecipitation (ChIP)

ChIP was performed using the ChIP Assay kit (Upstate). Briefly, 1–1.5×10^6^ fixed cells were sonicated for 10 sec at 30% amplitude to shear the chromatin (Branson Digital Sonifier 450, Crystal Electronics, On. Canada). Sonicated materials were immunoprecipitated using 2 µg mouse anti-p53 (DO-1), rabbit anti-H3Me2Lys9, rabbit anti-H3Me2Lys27 (Cell signaling), rabbit anti-Acetyl-H4, and anti-mouse IgG (Upstate) antibodies. Promoter fragments were amplified using primers to *xCT*, *Sod2* and *β major* chain as specified in Information S1.

### Animals

*Bmi1^+/−^* mice (in C57Bl/6J background) were a gift from M. Van Lohuizen (The Netherlands Cancer Institute, Amsterdam). Wild type C57Bl/6J inbred mice were purchased from Charles River Canada.

### Immunohistochemical analysis, quantitative real-time PCR, Western blot, measurement of ROS and lipid peroxidation levels

Technical details are provided in Information S1 [22].

### Statistical analysis

Statistical differences were analyzed using Student's *t*-test for unpaired samples. An analysis of variance (ANOVA) followed by the Dunnett test was used for multiple comparisons with one control group. In all cases, the criterion for significance (*P* value) was set as mentioned in the figures.

## Results

### Bmi1 is down-regulated in mouse cortical neurons during aging

**Figure 1:**
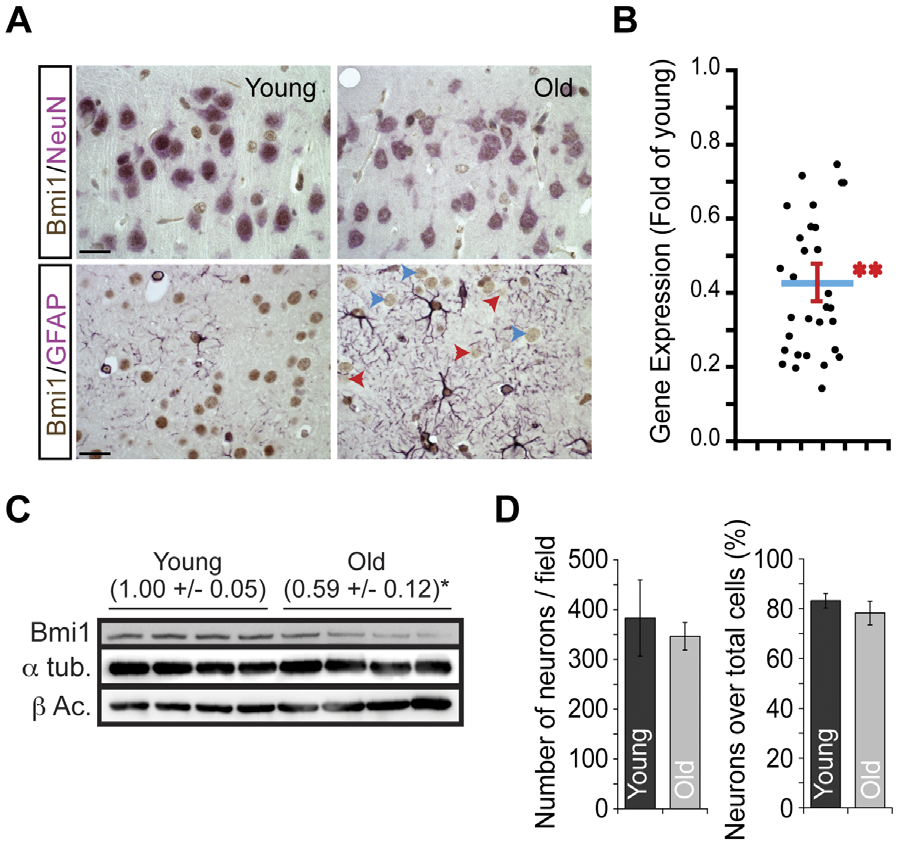
Bmi1 is down-regulated in the aging mouse brain. A) Coronal sections from the cerebral cortex were analyzed by IHC using Bmi1 and NeuN (top panels), or Bmi1 and GFAP (lower panels) antibodies. Bmi1 (brown) is highly expressed in NeuN^+^ (pink) neurons and its expression decreases with age, while it remains unchanged in GFAP^+^ (Pink) astrocytes. Note that in aged neurons, Bmi1 labeling was not uniform, with some neurons expressing Bmi1 at moderate levels (blue arrowheads), while others showing nearly undetectable level (red arrowheads). Scale bars; 20 µm. (B) The relative expression of *Bmi1* in cortices from young and old brains was analyzed by Q-PCR. Each point represents a comparison between one old and one young mouse cortex. The blue line represents the mean, and the red line represents the standard deviation (n = 15; **P<1.022E^−24^). (C)Young (40–55 days old) and old (21–26 months old) mice brain samples were analyzed by Western blot for Bmi1 expression. Protein loading was normalized using β-actin and α-tubulin. Data in brackets are levels of Bmi1 and are expressed as Mean ± s.d. (n = 4 brains per group; *P<0.05). (D)Thenumbers of neurons (NeuN+ cells) and non-neuronal cells (NeuN- cells) were counted in young and old mice brain slices. (left panel) Data were presented as absolute neuron number per cortical field. (right panel) Data were expressed as the percentage of neurons versus all cortical cells (neurons + non-neuronal cells). Results are Mean +/− s.d. (n = 3 brains per age, and counts were made on 4 to 9 slices per brain; P = 0.18).

To evaluate Bmi1 expression in neurons during aging, we analyzed sections of cerebral cortices from young (2 months) and old (21–26 months) mice using immunohistochemistry. We observed that Bmi1 expression decreased significantly in old NeuN^+^ cortical neurons, but not in GFAP^+^ astrocytes (Figure 1A). In old neurons, Bmi1 labeling was not uniform, with some neurons expressing Bmi1 at moderate levels, while others showing nearly undetectable levels (Figure 1A). Immunolabeling was not present in cortical sections from *Bmi1^−/−^* mice, confirming the specificity of the antibody (Figure S1). Referring to a previous report proposing that *Bmi1* is not down-regulated in the aging mouse brain [23], we measured *Bmi1* levels in a large cohort of young (n = 15) and old (n = 15) inbred mice. Using whole cerebral extracts and quantitative RT-PCR (Q-PCR), we found that *Bmi1* mRNA levels decrease by ∼60% in old cortices, thus suggesting reduced *Bmi1* transcription (Figure 1B). Interestingly, *Bmi1* expression levels were highly variable in the old age population, with some mice showing only 20% of *Bmi1* levels compared to young mice (Figure 1B). To confirm our observations at the protein levels, we perform Western blot analysis of whole cortices from young and old mice, which revealed a ∼40% reduction in Bmi1 levels in old mice (Figure 1C). To address the possibility that a variation in cell population numbers could underlies the observed reduction in Bmi1 levels, we counted and compared the number of NeuN^+^ neurons/cortical area or over the total number of cortical cell nuclei in young and old mice, but found no difference (Figure 1D).

**Figure 2:**
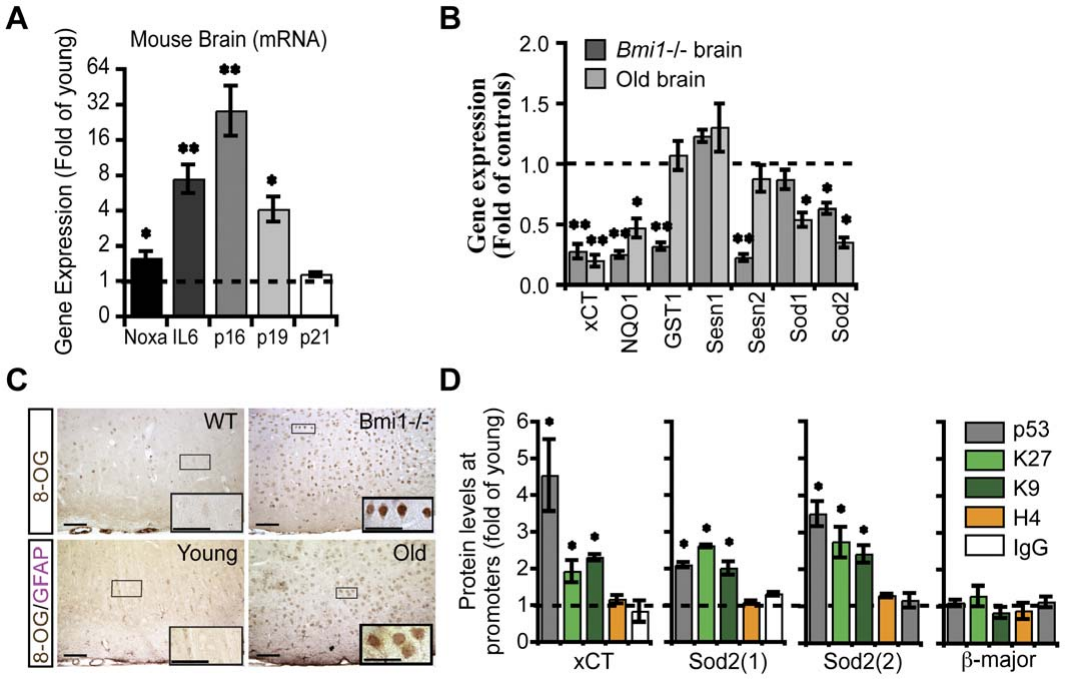
Antioxidant defenses are reduced in the aging mouse brain. (A) The relative expression of senescence-associated genes in cortices from young and old brains was analyzed by Q-PCR. Results are Mean ± s.d. (n = 3; *P<0.05; **P<0.01). The dashed line represents the basal gene expression level measured in young mice. (B) The relative expression of antioxidant genes in cortices from young and old brains, and from P25 *Bmi1^−/−^* and WT mice was analyzed by Q-PCR. The dashed line represents the basal gene expression level measured in young compared to old and to WT compared to *Bmi1^−/−^* mice. Results are Mean ± s.d. (n = 3; *P<0.05; **P<0.01). (C) Coronal sections from the cerebral cortex of young and old mice, and of P25 WT and *Bmi1^−/−^* mice were labeled with antibodies against 8-oxo-guanine (8-OG; brown) and GFAP (pink). Note the increase in 8-oxo-guanine labeling in neurons from old and *Bmi1^−/−^* mice compared to respective controls. Scale bars; 50 µm. (D) ChIP analysis of young and old brains revealing accumulation of p53 and heterochromatin marks (histone H3 K27^me2^ or H3 k9^me2^) at the *xCT*, *Sod1* and *Sod2* promoters in old brains. Antibodies against acetylated histone H4 and IgG were used as control. The *β-major* promoter region of *globin* was use as negative control. Results are Mean ± s.d. (n = 3; *P<0.05).

*p16^Ink4a^* and *p19^Arf^* are the main targets of Bmi1 transcriptional repressive activity in most of cell types, including cortical neurons [18], [20]. Expression of *p16^Ink4a^* and *p19^Arf^* was increased concomitantly with *Bmi1* reduction in old brains (Figure 2A). The similarity between normal brain aging and *Bmi1* genetic deficiency also extended to *Noxa*, a p53-regulated gene involved in apoptosis, and *IL-6*, which is involved in inflammation and inflammatory senescence (Figure 2A) [24], [25]. Infection of cultured *Bmi1^−/−^* neurons with a dominant negative adenovirus against p53 (DNp53) could restore *IL-6* expression to normal levels, but not those of *p16^Ink4a^* and *p19^Arf^* (Figure S2), which are primary Bmi1 targets [18], [20], [26]. To further seek for possible gene expression pattern resemblances between brain aging and *Bmi1* genetic deficiency, we compared the expression of AOR genes in cortices of both young and old mice, and of post-natal day (P) 25 WT and *Bmi1^−/−^* mice. Notably, several genes involved in ROS scavenging were down-regulated in old brains, revealing a general decrease in antioxidant defenses (Figure 2B). Common down-regulated genes between *Bmi1^−/−^* brains and old brains were xCT, NQO1 and Sod2 (Figure 2B). These genes are also down-regulated in cultured *Bmi1^−/−^* neurons [20]. To commensurate the above observations, we compared the accumulation of oxidative damages between young and old brains, and between WT and *Bmi1^−/−^* P25 brains or cultured neurons. Concentrations of oxidized lipids were increased in old brains (1.40±0.05, *P*<0.05, n = 3) compared to the young samples, similarly as between *Bmi1^−/−^* (1.43±0.03, *P*<0.05, n = 3) and WT brains or *Bmi1^−/−^* (2.54±0.12, *P*<0.01, n = 3) and WT e18.5 neurons. Concomitant with this finding, accumulation of oxidized genomic DNA bases (8-hydroxydeoxyguanosine) was elevated in neurons of old and P25 *Bmi1^−/−^* brains (Figure 2C) [27], [28]. In cortical neurons, 8-oxoguanine lesions were found in both nuclear and mitochondrial DNA (Figure 2C). This latter finding implies increased oxidative DNA damages in cortical neurons from old and P25 *Bmi1^−/−^* mice owing to reduced antioxidant defenses. This primary defect may be combined with a reduction in 8-oxoguanine DNA glycosylase (OGG1) activity or level [29], [30], [31]. It was previously showed that p53 proteins levels progressively increase in the aging rat brain [28]. Furthermore, p53 protein can associate with the promoters of AOR genes such as those encoding glutathione S-transferase alpha 1 (GSTα1), NAD(P)H quinone oxidoreductase (NQO1) and xCT. Upon damage, activated p53 can repress the transcription of these genes and interfere with the antioxidant defense system normally activated by the Nrf2 transcription factor [32]. To test for a possible mechanism of AOR genes downregulation in the aging brain, we compared young and aged brains for p53 accumulation at the *xCT* and *Sod2* promoters by ChIP. We found that p53 was enriched at the *xCT* promoter and in two distinct *Sod2* promoter regions in aged brains (Figure 2D). Interestingly, this correlated with a repressed chromatin state, as shown by the accumulation of heterochromatin marks at these promoters, similarly as found in cultured *Bmi1^−/−^* neurons (Figure 2D) [20].

### BMI1 is down-regulated in the human CNS during aging

**Figure 3:**
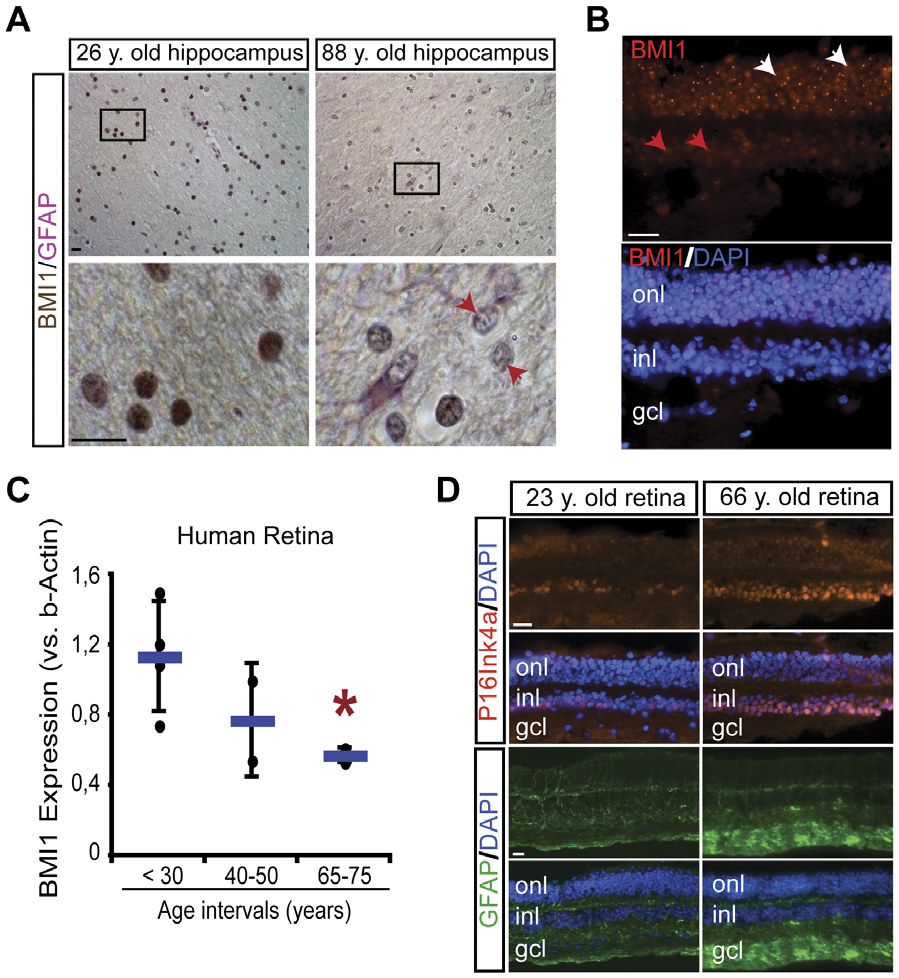
BMI1 is down-regulated in the aging human brain and retina. (A) Immunohistochemistry on human brain (hippocampus) sections using anti-Bmi1 (brown) and anti-GFAP (pink) antibodies. BMI1 is expressed in neurons, but not in GFAP+ astrocytes, and expression is highly reduced in old brain neurons. Note the virtual absence of BMI1 labeling in some neurons (red arrowheads). Scale bars; 20 µm. (B) Immunofluorescence analysis of BMI1 expression in the human retina (23 years old, frozen sections). BMI1 is highly expressed in human photoreceptors (white arrowheads), which cell body lies in the outer nuclear layer (ONL), while its expression is weaker in neurons of the inner nuclear (INL) and ganglion cell (GCL) layers (red arrowheads). Scale bars; 20 µm. (C) Human retina samples were analyzed by Western blot for BMI1 expression and protein content was normalized using *β*-actin. BMI1 protein levels are reduced in old retinas (65–75 years). Results are Mean ± s.d. (n = 2–5 retinas per group; *P<0.05). (D)Immunofluorescence analysis of GFAP and P16^INK4A^ expression in young and old human retinas. Note increased GFAP and P16^INK4A^ immunoreactivity in the old retinas. Scale bars; 20 µm.

To test if age-dependent Bmi1 down-regulation was restricted to mice, we analyzed BMI1 expression in young and old human brains by immunohistochemistry. We observed that BMI1 expression was reduced in hippocampal neurons of old brains (Figure 3A). In contrast to the observations found in mice, BMI1 is not expressed in normal human astrocytes, even in young brains [20]. We previously reported on Bmi1 expression in post-mitotic neurons of the mouse retina. Using immunofluorescence, we analyzed BMI1 expression in the adult human retina. We observed robust BMI1 immunoreactivity in the nucleus of photoreceptors and weaker expression in neurons of the inner nuclear and ganglion cell layers (Figure 3B). The peculiar expression of BMI1 in some photoreceptors was distinct from classical polycomb bodies staining (Figure 3B). To quantify for possible BMI1 down-regulation in the aging human retina, we performed Western blot analysis using young (4–30 years old), middle-aged (40 and 50 years old) and old (65–75 years old) human retinas. BMI1 protein levels were variable from sample to sample, especially in the young and middle-aged groups, in agreement with previous studies [1]. Nonetheless, BMI1 levels were consistently lower in old human retinas (Figure 3C). Linear regression analysis suggests a stepwise reduction in BMI1 levels, and where expression between 85–95 years of age could be, on average, less than 30% of what is found in young individuals. However, due to samples limitations, BMI1 expression in retinas older than 75 years could not be tested in this study. Reactive gliosis in the retina and in the brain is observed in neurodegenerative diseases, CNS lesions and during aging and can be visualize by GFAP immunoreactivity. P16^INK4A^ represents a biomarker of tissue aging and is a target of Bmi1 repressive activity [23], [33], [34]. Consistently, we observed increased GFAP and P16^INK4A^ immunoreactivity in sections of aged human retinas (Figure 3D), similarly as observed in the retina and the brain of *Bmi1^−/−^* mice at P25 [20], [35].

### Bmi1 over-expression is neuroprotective and activates antioxidant defenses

**Figure 4:**
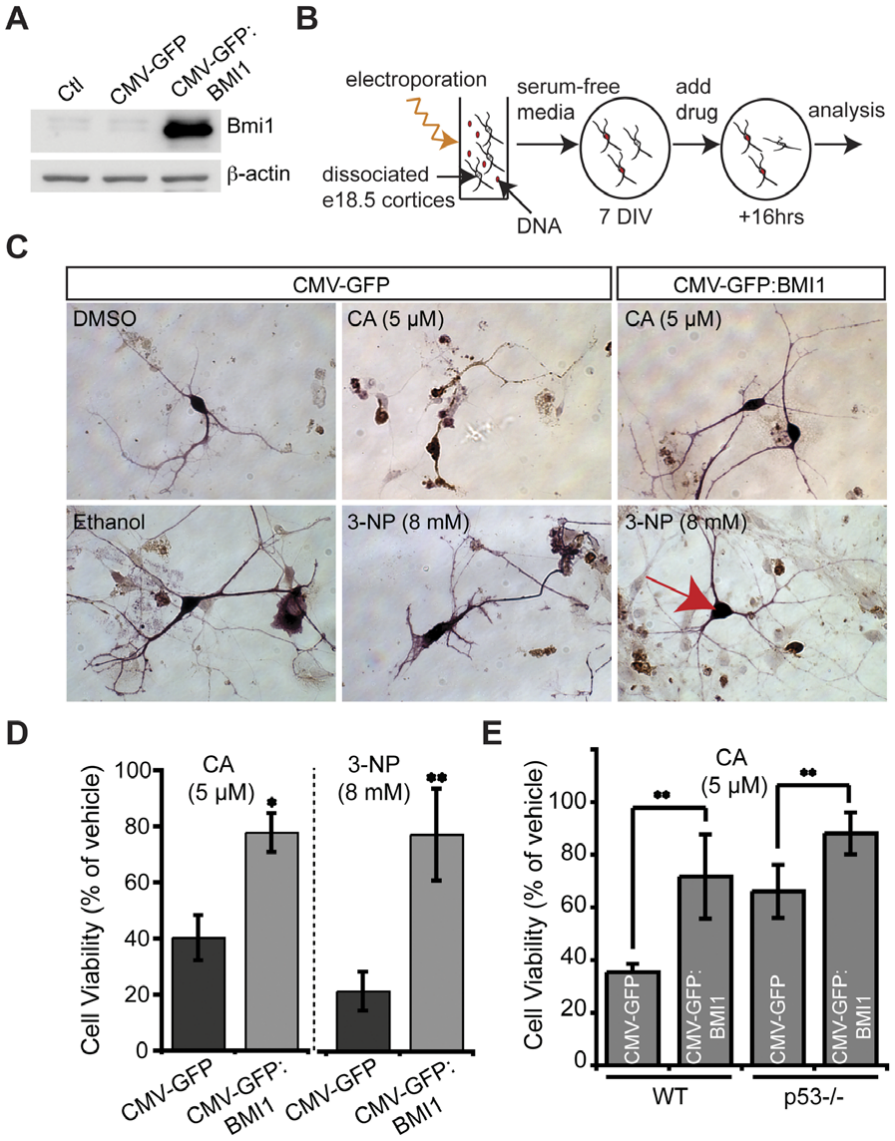
BMI1 is highly neuroprotective against topoisomerase I inhibition and mitochondrial poisoning. (A) Empty plasmid vectors (CMV-GFP) or human BMI1-carrying plasmid (CMV-GFP: BMI1) were transfected in 293FT cells and lysates were analyzed 72 hours later for Bmi1 expression by Western blot. β-actin was used as internal control for normalization of protein loading. Non-transfected cells were used as control (Ctl) for endogenous Bmi1 expression. (B) Experimental scheme showing the procedure used to electroporate plasmid vectors in primary neuronal cultures from e18.5 WT mouse embryo cortices. (C) After 7 days i*n vitro* (DIV), electroporated neurons were exposed to CA, 3-NP or their respective vehicles. 16 hours later, cultures were stained for apoptosis induction (caspase-3 in brown) and expression of GFP (in pink), in order to distinguish neurons carrying or not the transgene. (D) Cell viability was assessed in cultures photographed in (C) as the percentage of GFP^+^/Caspase-3^−^ cells *versus* total GFP^+^ cells. Results are Mean ± s.d. (n = 3; *P<0.05; **P<0.001). (E) After 7 DIV, electroporated WT and *p53^−/−^* neurons were exposed to CA or vehicle (DMSO) and analyzed after 16 hours as described in (C). Results are Mean ± s.d. (n = 3; **P<0.001).

To test if increasing Bmi1 expression could protect neurons from damage-induced cell death, we generated a plasmid expressing the complete human BMI1 cDNA (Figure 4A). This expression vector was electroporated in e18.5 WT cortical neurons that were thereafter exposed or not to neuronal toxins (Figure 4B). Using immunohistochemistry, neurons viability was determined by the absence of activated caspase-3 and the maintenance of long axons and dendrites presenting a rich arborization, similarly as observed in vehicle-treated neurons. When neurons were exposed to 5 µM of camptothecin (CA), a DNA topoisomerase I inhibitor that leads to single-strand DNA breaks formation and p53-mediated neuronal apoptosis, only ∼40% of control-GFP electroporated neurons were viable after 16 hours [24], [36], [37], [38], [39]. In contrast, ∼80% of GFP/BMI1-transfected neurons remained viable after this time point (Figure 4C and D). These data revealed that BMI1 is required and sufficient to block CA-induced apoptosis cell autonomously. To test if BMI1 over-expression was neuroprotective against a mitochondrial toxin, we treated neurons with 8 mM of 3-nitroproprionic acid (3-NP), a succinate dehydrogenase inhibitor that blocks complex II of the mitochondria respiratory chain [40], [41]. 3-NP treatment induced caspase-3-dependent (apoptosis) and caspase-3-independent (vacuolization) neuronal cell death (Figure 4C). At this high concentration, only ∼21% of GFP-electroporated neurons remained viable after 16 hours. In contrast, ∼77% of GFP/BMI1-transfected neurons remained viable at this concentration, showing that BMI1 over-expression confers cell autonomous protection against 3-NP toxicity (Figure 4C and D). We previously reported that p53 protein levels were increased in *Bmi1^−/−^* neurons and that the major effects of *Bmi1* genetic deficiency were mediated by p53 pro-apoptotic and pro-oxidant activity. To test part of the mechanism involved in BMI1 neuroprotective activity, we over-expressed BMI1 by DNA electroporation in WT and *p53^−/−^* neurons treated or not with 5 µM of CA. We observed that although *p53^−/−^* neurons were highly resistant to CA treatment compared to WT neurons, this effect was further enhanced when BMI1 was over-expressed (Figure 4E).

**Figure 5:**
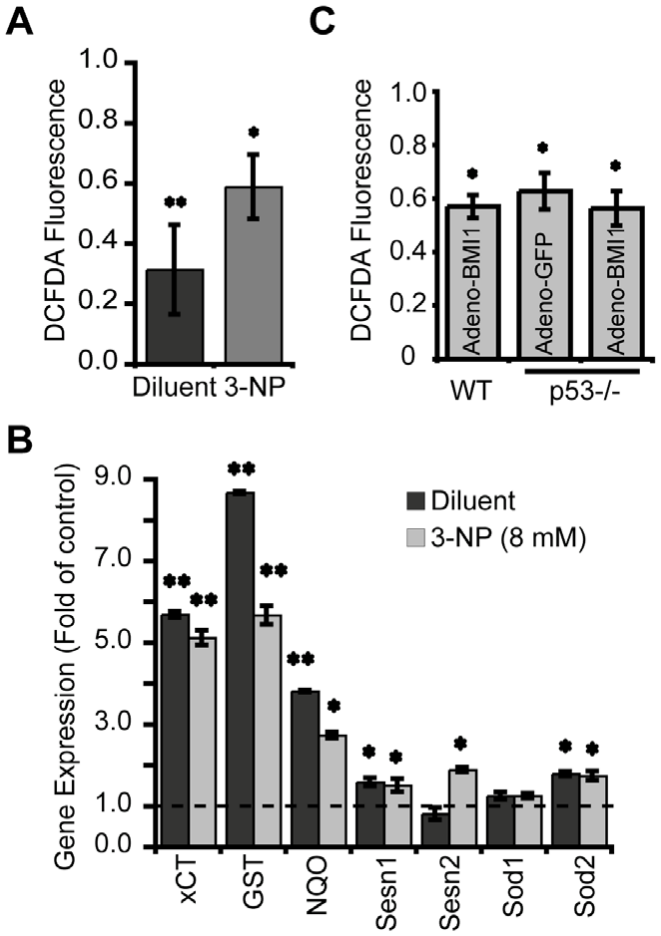
BMI1 over-expression activates AOR genes and suppresses ROS. (A and B) e18.5 WT neurons were infected with BMI1-expressing (Adeno-BMI1) or GFP-expressing (Adeno-GFP) adenoviruses. 48 hours post-infection, neurons were treated or not with 8 mM of 3-NP and analyzed 6 hours later. (A) ROS levels were measured with the fluorescent dye DCFDA. Data were normalized to the protein contents and expressed as fold change relative to levels in Adeno-GFP-infected cells. Results are Mean ± s.d. (n = 3 independent cultures; *P<0.05; **P<0.01). (B) relative gene expression was analyzed by Real-Time PCR. Data are normalized to levels found in adeno-GFP-infected neurons that were set at 1 (dashed line). Results are Mean ± s.d. (n = 3 independent cultures; *P<0.05; **P<0.01). (C) e18.5 WT or *p53−/−* neurons were infected with adenoviruses and analyzed for ROS levels as in (A). Data were expressed as fold change relative to levels in Adeno-GFP-infected WT neurons. Results are Mean ± s.d. (n = 3 independent cultures; *P<0.05).

To address if BMI1 activity correlated with ROS modulation and induction of AOR genes expression, a BMI1-expressing adenovirus was used to infect cortical neurons in culture. 48 hours post-infection, neurons were treated or not with 8 mM of 3-NP and analyzed 6 hours later for ROS concentrations. We found that BMI1 over-expression reduced ROS concentrations in untreated neurons (Figure 5A). This biochemical effect was reflected by an increase in phase II genes (i.e. xCT, NQO1 and GST-1α) expression by an order of 3–9 fold and induction of Sestrin1 and Sod2 by an order of 1.5–2 fold (Figure 5B). Upon 3-NP exposure, we also observed a reduction in ROS concentrations in BMI1-overexpressing neurons relative to GFP-infected neurons, and this was again reflected by activation of antioxidant genes (Figure 5A and B). We concluded that in gain-of-function experiments, BMI1 can reduce ROS levels and over-activate antioxidant defenses. Notably, *p53^−/−^* neurons infected with the BMI1-expressing adenovirus or control adenovirus (GFP-only) showed nearly identical ROS concentrations, and these were comparable to ROS concentrations found in WT neurons infected with the BMI1-expressing adenovirus (Figure 5C). These later results suggest that parts of BMI1 neuroprotective activities may be *p53*-independent and not mediated through regulation of ROS concentrations.

## Discussion

Herein, we demonstrated that the Polycomb group gene Bmi1 is down-regulated in the brain and the retina of aged mice and humans. Moreover, numerous aspects of brain aging, but not all, are recapitulated in the progeroid phenotype of *Bmi1^−/−^* mice, suggesting a model linking Bmi1 deficiency and unregulated p53 activity to reduced antioxidant defenses in the aging brain. On the other hand, we presented evidences that BMI1 over-expression is neuroprotective, can activate AOR genes, and can suppress ROS.

In the central nervous system of mammals, peroxisome proliferator-activated receptor gamma coactivator-1α (PGC-1α) has been shown to be a major inducer of the antioxidant defense system. Consistent with this, dopaminergic neurons from PGC-1α-null mice are hypersensitive to the mitochondrial complex I inhibitor MPTP, and PGC-1α over-expression is neuroprotective against paraquat and H_2_O_2_ [42]. Likewise, Nrf2 over-expression in astrocytes can protect neurons in a non-cell autonomous manner from 3-NP toxicity, while Sirt1 over-expression can delay axonal degeneration and counteract neurodegeneration in a mouse model of Alzheimer disease [40], [41], [43], [44]. However, with the exception of a modest reduction in *Nrf1* mRNA levels, the expression of *PGC-1*α, *Sirt1* and *Nrf2* was unaltered at the mRNA and protein levels in *Bmi1^−/−^* mice [20]. Hence, the neuroprotective effects of BMI1 over-expression against CA and 3-NP toxicity are unlikely to be mediated through modulation of these factors. Rather, we propose that most of these effects are mediated through repression of p53 activity. In support of this 1) AOR genes expression profile of BMI1-overexpressing neurons is similar to that of *p53*-null neurons, and 2) p53 is the central mediator of CA-induced apoptosis in neurons [20], [24], [36], [37], [38], [39]. On the other hand, we also showed that BMI1 over-expression in *p53^−/−^* neurons confers increased neuronal survival after CA treatment, but not reduction in ROS concentrations. Our finding suggests that BMI1 neuroprotective activities are not only mediated via p53-dependent and ROS-dependent pathways. Although numerous possibilities exist, this could be achieved through BMI1 repression of alternate tumor suppressors and/or improved DNA damage response and repair [45], [46], [47]. Notably, the mechanism by which Bmi1 regulates p53 activity remains elusive, although repression of the p19^Arf^/Mdm2/p53 pathway through direct transcriptional repression of *p19^Arf^* by Bmi1 remains a possibility.

**Figure 6:**
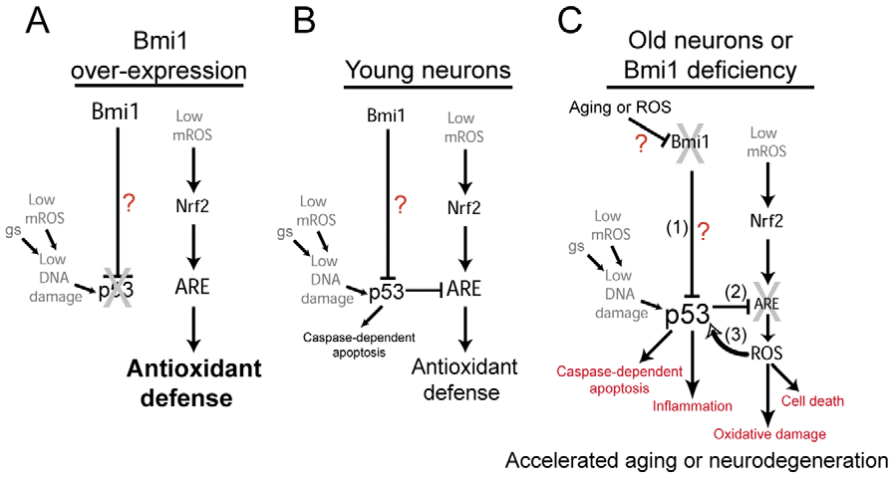
Bmi1 deficiency during aging influences neurons resistance to genotoxic stresses and mitochondrial dysfunctions. Proposed model of Bmi1 function in neurons: (A) When over-expressed, Bmi1 represses p53 activity by an unknown mechanism, leading to complete inhibition of p53 pro-apoptotic and pro-oxidant activities and supra-activation of the antioxidant defense system. (B) In young neurons, where Bmi1 expression is robust, Bmi1 partially represses p53 activity, thus allowing modulation of p53-mediated apoptosis and repression of antioxidant response elements (ARE). These elements are present in antioxidant-coding genes activated by the Nrf2 transcription factor. (C) In aging neurons, where Bmi1 expression becomes deficient, p53 is activated (1), leading to induction of apoptosis and inflammation, and in transcriptional repression of antioxidant-coding genes (2). Elevated mitochondrial reactive oxygen species (mROS) concentrations ultimately induce damages to lipids and DNA, which further activate p53 (3), resulting in the formation of a vicious circle. This situation renders old neurons particularly more vulnerable to genotoxic stresses (gs) and mitochondrial dysfunctions. This model is based on data from the present work, and those published previously [20].

The contexts of *Bmi1* deficiency in mice (which leads to a severe and early lethal pathology) and of physiological aging are completely different. Hence, it is most unexpected that *Bmi1* down-regulation in neurons during brain physiological aging leads to a molecular phenotype that resembles *Bmi1* genetic deficiency. The apparent conservation of the Bmi1-p53 antioxidant relationship during physiological aging is thus an important finding that brings new knowledge on the mechanisms regulating the process of physiological aging in the central nervous system. Our previous findings provided a molecular mechanism to explain why *Bmi1*-null neurons age prematurely and are hypersensitive to various stressors [20]. Considering Bmi1 down-regulation in the aging central nervous system, this may also explain why aged neurons are more sensitive to various insults. Cellular and DNA damage produced by mitochondrial ROS have been proposed to be the causal factor of cellular and organismal aging [1], [3], [7]. Herein we observed that p53 accumulates at AOR gene promoters in the aging mouse brain, correlating with reduced AOR genes expression. This suggests that deficient BMI1 expression in the human brain may contribute to reduce antioxidant defenses, leading to accumulation of oxidative damage and neuronal dysfunction. Although controversial, neuroinflammation is potentially a driving force of normal brain aging and neurodegenerative diseases. The cytokine IL-6 is a biomarker of brain aging, and high levels of IL-6 are found in senescent cells and Alzheimer disease brains [25], [48]. We found that old and *Bmi1^−/−^* brains expressed high levels of IL-6, and that this was p53-dependent in cultured *Bmi1^−/−^* neurons. These observations suggest that Bmi1 deficiency during aging could amplify p53 activity and thus accelerate the process of normal brain aging and age-related neurological diseases in readily susceptible individuals (see proposed model in Figure 6). Finally, while increased p53 activity in aging neurons may be in part explained by the loss of Bmi1 expression, the age-associated mechanism leading to Bmi1 down-regulation remains to be investigated. Although this mechanism apparently operates at the transcriptional level, as reported for other genes in the aging brain [1], age-dependent *Bmi1* mRNA degradation through specific miRNA activity is possible [49]. Also, it cannot be excluded that activated p53 represses Bmi1 transcription, although such evidences do not exist yet. At last, Bmi1 protein may be also preferentially targeted for degradation in aging neurons [50], [51], [52]. In old mice, Bmi1 expression declined only in neurons, not in astrocytes. Notably, only astrocytes have the potential to generate tumors. These data indicate that down-regulation of the Bmi1 oncogene in neurons during aging is apparently not a general response to prevent cancer (at least in mice). Rather, it may represent an epigenetic change related to normal brain aging [1]. Taken together, our work suggests that targeted pharmaceutical stimulation of *Bmi1* expression in aging neurons could prevent accumulation of age-associated oxidative damages, protect against apoptosis and possibly counteract the progression of neurodegenerative diseases.

## Supporting Information

Figure S1 **Coronal sections of WT and** ***Bmi1^−/−^*** **mice brains were analyzed by IHC using Bmi1 (brown) and NeuN (pink) antibodies.** Note that there is no Bmi1 staining in the mutant sample. Scale bars; 50 µm.
(TIF)

Figure S2 **Cultured WT and** ***Bmi1^−/−^*** **neurons were infected or not with an adenovirus expressing a dominant-negative form of p53, and gene expression levels were analyzed by qPCR 48 hours later.** P53 inhibition restored *IL-6* expression, but not *p16^Ink4a^* and *p19^Arf^* expression, to levels comparable to those found in WT neurons. Data are normalized to hprt expression levels. Results are Mean +/− s.d. (n = 4 independent cultures; **P<0.01 when compared to Bmi1−/−).
(TIF)

Information S1 **Contains the description of supplementary methods.**
(DOC)

## References

[1] Lu T, Pan Y, Kao SY, Li C, Kohane I (2004). Gene regulation and DNA damage in the ageing human brain.. Nature.

[2] Best BP (2009). Nuclear DNA damage as a direct cause of aging.. Rejuvenation Res.

[3] Halliwell B (2006). Oxidative stress and neurodegeneration: where are we now?. J Neurochem.

[4] Bielas JH, Heddle JA (2000). Proliferation is necessary for both repair and mutation in transgenic mouse cells.. Proc Natl Acad Sci U S A.

[5] Bielas JH, Heddle JA (2004). Quiescent murine cells lack global genomic repair but are proficient in transcription-coupled repair.. DNA Repair (Amst).

[6] Rass U, Ahel I, West SC (2007). Defective DNA repair and neurodegenerative disease.. Cell.

[7] Balaban RS, Nemoto S, Finkel T (2005). Mitochondria, oxidants, and aging.. Cell.

[8] Lin MT, Beal MF (2006). Mitochondrial dysfunction and oxidative stress in neurodegenerative diseases.. Nature.

[9] Harman D (1956). Aging: a theory based on free radical and radiation chemistry.. J Gerontol.

[10] Schriner SE, Linford NJ, Martin GM, Treuting P, Ogburn CE (2005). Extension of murine life span by overexpression of catalase targeted to mitochondria.. Science.

[11] Orr WC, Sohal RS (1994). Extension of life-span by overexpression of superoxide dismutase and catalase in Drosophila melanogaster.. Science.

[12] Sentman ML, Granstrom M, Jakobson H, Reaume A, Basu S (2006). Phenotypes of mice lacking extracellular superoxide dismutase and copper- and zinc-containing superoxide dismutase.. J Biol Chem.

[13] Unlu ES, Koc A (2007). Effects of deleting mitochondrial antioxidant genes on life span.. Ann N Y Acad Sci.

[14] Valk-Lingbeek ME, Bruggeman SW, Van Lohuizen M (2004). Stem cells and cancer; the polycomb connection.. Cell.

[15] Buchwald G, van der Stoop P, Weichenrieder O, Perrakis A, van Lohuizen M (2006). Structure and E3-ligase activity of the Ring-Ring complex of polycomb proteins Bmi1 and Ring1b.. Embo J.

[16] Cao R, Tsukada Y, Zhang Y (2005). Role of Bmi-1 and Ring1A in H2A ubiquitylation and Hox gene silencing.. Mol Cell.

[17] Li Z, Cao R, Wang M, Myers MP, Zhang Y (2006). Structure of a Bmi-1-Ring1B polycomb group ubiquitin ligase complex.. J Biol Chem.

[18] Jacobs JJ, Kieboom K, Marino S, DePinho RA, van Lohuizen M (1999). The oncogene and Polycomb-group gene bmi-1 regulates cell proliferation and senescence through the ink4a locus.. Nature.

[19] van der Lugt NM, Domen J, Linders K, van Roon M, Robanus-Maandag E (1994). Posterior transformation, neurological abnormalities, and severe hematopoietic defects in mice with a targeted deletion of the bmi-1 proto-oncogene.. Genes Dev.

[20] Chatoo W, Abdouh M, David J, Champagne MP, Ferreira J (2009). The polycomb group gene Bmi1 regulates antioxidant defenses in neurons by repressing p53 pro-oxidant activity.. J Neurosci.

[21] Chatoo W, Abdouh M, Bernier G (2011). P53 pro-oxidant activity in the central nervous system: implication in aging and neurodegenerative diseases.. Antioxid Redox Signal.

[22] Buege JA, Aust SD (1978). Microsomal lipid peroxidation.. Methods Enzymol.

[23] Krishnamurthy J, Torrice C, Ramsey MR, Kovalev GI, Al-Regaiey K (2004). Ink4a/Arf expression is a biomarker of aging.. J Clin Invest.

[24] Cregan SP, Arbour NA, Maclaurin JG, Callaghan SM, Fortin A (2004). p53 activation domain 1 is essential for PUMA upregulation and p53-mediated neuronal cell death.. J Neurosci.

[25] Rodier F, Coppe JP, Patil CK, Hoeijmakers WA, Munoz DP (2009). Persistent DNA damage signalling triggers senescence-associated inflammatory cytokine secretion.. Nat Cell Biol.

[26] Ferbeyre G, de Stanchina E, Lin AW, Querido E, McCurrach ME (2002). Oncogenic ras and p53 cooperate to induce cellular senescence.. Mol Cell Biol.

[27] Markesbery WR, Lovell MA (2006). DNA oxidation in Alzheimer's disease.. Antioxid Redox Signal.

[28] Dorszewska J, Adamczewska-Goncerzewicz Z (2004). Oxidative damage to DNA, p53 gene expression and p53 protein level in the process of aging in rat brain.. Respir Physiol Neurobiol.

[29] Lovell MA, Markesbery WR (2007). Oxidative DNA damage in mild cognitive impairment and late-stage Alzheimer's disease.. Nucleic Acids Res.

[30] Shao C, Xiong S, Li GM, Gu L, Mao G (2008). Altered 8-oxoguanine glycosylase in mild cognitive impairment and late-stage Alzheimer's disease brain.. Free Radic Biol Med.

[31] Tian F, Tong TJ, Zhang ZY, McNutt MA, Liu XW (2009). Age-dependent down-regulation of mitochondrial 8-oxoguanine DNA glycosylase in SAM-P/8 mouse brain and its effect on brain aging.. Rejuvenation Res.

[32] Faraonio R, Vergara P, Di Marzo D, Pierantoni MG, Napolitano M (2006). p53 suppresses the Nrf2-dependent transcription of antioxidant response genes.. J Biol Chem.

[33] Silver J, Miller JH (2004). Regeneration beyond the glial scar.. Nat Rev Neurosci.

[34] Sherr CJ (2001). The INK4a/ARF network in tumour suppression.. Nat Rev Mol Cell Biol.

[35] Zencak D, Lingbeek M, Kostic C, Tekaya M, Tanger E (2005). Bmi1 loss produces an increase in astroglial cells and a decrease in neural stem cell population and proliferation.. J Neurosci.

[36] Cregan SP, MacLaurin JG, Craig CG, Robertson GS, Nicholson DW (1999). Bax-dependent caspase-3 activation is a key determinant in p53-induced apoptosis in neurons.. J Neurosci.

[37] Slack RS, Belliveau DJ, Rosenberg M, Atwal J, Lochmuller H (1996). Adenovirus-mediated gene transfer of the tumor suppressor, p53, induces apoptosis in postmitotic neurons.. J Cell Biol.

[38] Xiang H, Hochman DW, Saya H, Fujiwara T, Schwartzkroin PA (1996). Evidence for p53-mediated modulation of neuronal viability.. J Neurosci.

[39] Xiang H, Kinoshita Y, Knudson CM, Korsmeyer SJ, Schwartzkroin PA (1998). Bax involvement in p53-mediated neuronal cell death.. J Neurosci.

[40] Calkins MJ, Jakel RJ, Johnson DA, Chan K, Kan YW (2005). Protection from mitochondrial complex II inhibition in vitro and in vivo by Nrf2-mediated transcription.. Proc Natl Acad Sci U S A.

[41] Shih AY, Imbeault S, Barakauskas V, Erb H, Jiang L (2005). Induction of the Nrf2-driven antioxidant response confers neuroprotection during mitochondrial stress in vivo.. J Biol Chem.

[42] St-Pierre J, Drori S, Uldry M, Silvaggi JM, Rhee J (2006). Suppression of reactive oxygen species and neurodegeneration by the PGC-1 transcriptional coactivators.. Cell.

[43] Araki T, Sasaki Y, Milbrandt J (2004). Increased nuclear NAD biosynthesis and SIRT1 activation prevent axonal degeneration.. Science.

[44] Kim D, Nguyen MD, Dobbin MM, Fischer A, Sananbenesi F (2007). SIRT1 deacetylase protects against neurodegeneration in models for Alzheimer's disease and amyotrophic lateral sclerosis.. Embo J.

[45] Abdouh M, Facchino S, Chatoo W, Balasingam V, Ferreira J (2009). BMI1 sustains human glioblastoma multiforme stem cell renewal.. J Neurosci.

[46] Facchino S, Abdouh M, Chatoo W, Bernier G (2010). BMI1 confers radioresistance to normal and cancerous neural stem cells through recruitment of the DNA damage response machinery.. J Neurosci.

[47] Ismail IH, Andrin C, McDonald D, Hendzel MJ (2010). BMI1-mediated histone ubiquitylation promotes DNA double-strand break repair.. J Cell Biol.

[48] Wyss-Coray T (2006). Inflammation in Alzheimer disease: driving force, bystander or beneficial response?. Nat Med.

[49] Godlewski J, Nowicki MO, Bronisz A, Williams S, Otsuki A (2008). Targeting of the Bmi-1 oncogene/stem cell renewal factor by microRNA-128 inhibits glioma proliferation and self-renewal.. Cancer Res.

[50] Kim J, Hwangbo J, Wong PK (2011). p38 MAPK-Mediated Bmi-1 down-regulation and defective proliferation in ATM-deficient neural stem cells can be restored by Akt activation.. PLoS One.

[51] Kim J, Wong PK (2009). Oxidative stress is linked to ERK1/2-p16 signaling-mediated growth defect in ATM-deficient astrocytes.. J Biol Chem.

[52] Sahasrabuddhe AA, Dimri M, Bommi PV, Dimri GP (2011). betaTrCP regulates BMI1 protein turnover via ubiquitination and degradation.. Cell Cycle.

