# Supplementary figures and images for: Bmi1 Is Down-Regulated in the Aging Brain and Displays Antioxidant and Protective Activities in Neurons
Source: PLoS One. 2012 Feb 23;7(2):e31870. doi: 10.1371/journal.pone.0031870 (PMC3285640; doi:10.1371/journal.pone.0031870)

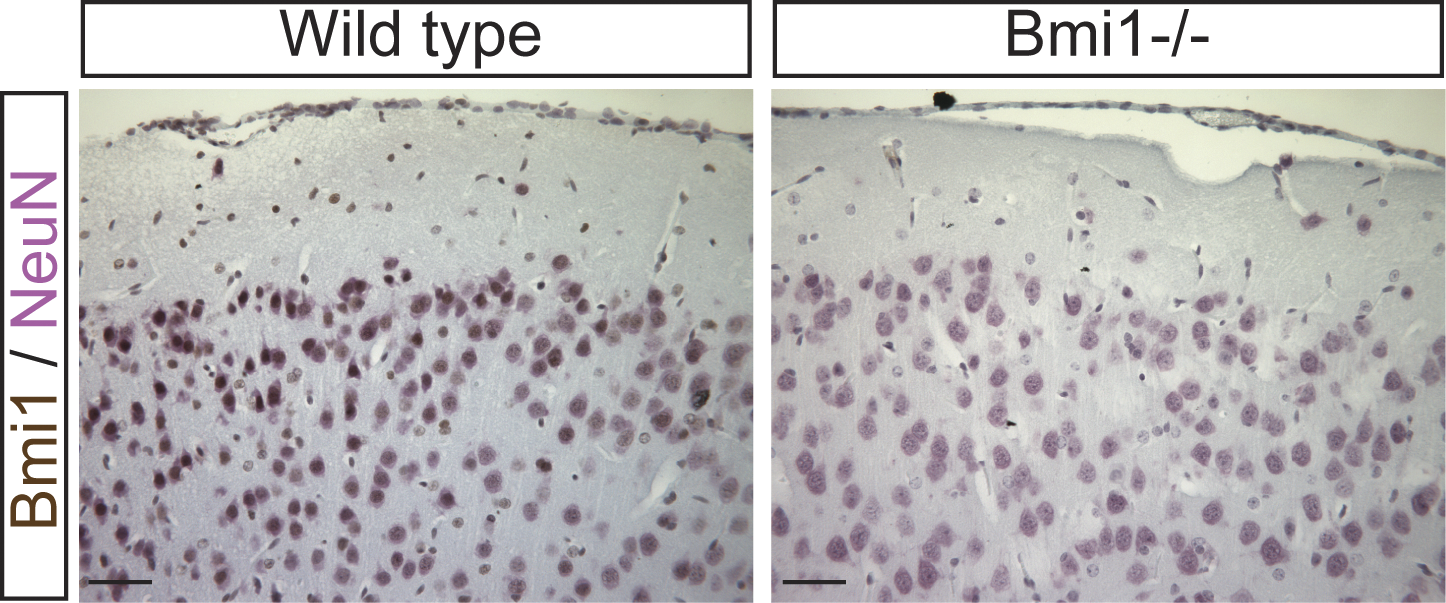

Supplement: Figure S1 — Coronal sections of WT and Bmi1−/− mice brains were analyzed by IHC using Bmi1 (brown) and NeuN (pink) antibodies. Note that there is no Bmi1 staining in the mutant sample. Scale bars; 50 µm. (TIF) [file pone.0031870.s001.tif]

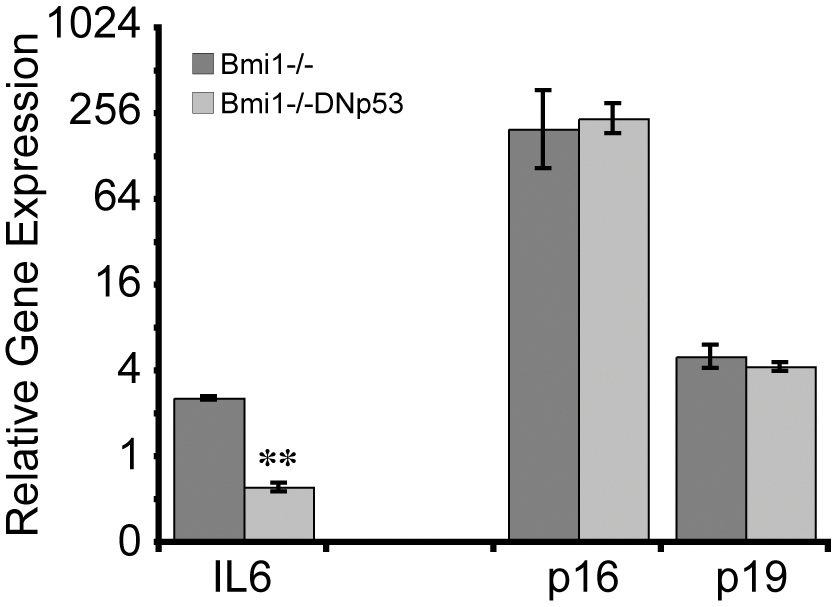

Supplement: Figure S2 — Cultured WT and Bmi1−/− neurons were infected or not with an adenovirus expressing a dominant-negative form of p53, and gene expression levels were analyzed by qPCR 48 hours later. P53 inhibition restored IL-6 expression, but not p16Ink4a and p19Arf expression, to levels comparable to those found in WT neurons. Data are normalized to hprt expression levels. Results are Mean +/− s.d. (n = 4 independent cultures; **P<0.01 when compared to Bmi1−/−). (TIF) [file pone.0031870.s002.tif]
