# Supplementary material for: Bmi1 Is Down-Regulated in the Aging Brain and Displays Antioxidant and Protective Activities in Neurons
Source: PLoS One. 2012 Feb 23;7(2):e31870. doi: 10.1371/journal.pone.0031870 (PMC3285640; doi:10.1371/journal.pone.0031870)
Supplement: Information S1 — Contains the description of supplementary methods. (DOC) [file pone.0031870.s003.doc]

**Supporting Information S1**

**BMI1 is down-regulated in the aging brain and displays robust neuroprotective and antioxidant activities**

Mohamed Abdouh^1¶^, Wassim Chatoo^1¶^, Jida El Hajjar^1^, Jocelyn David^1^, José Ferreira^2^, and Gilbert Bernier^1, 3 *^

^1^ Developmental Biology Laboratory, Hôpital Maisonneuve-Rosemont, Montréal, Canada

^2^ Department of Pathology, Hôpital Maisonneuve-Rosemont, Montréal, Canada

^3^ Department of Ophthalmology, Université de Montréal, Montréal, Canada

^¶^ Equal contribution

*Corresponding author:

Tel: 514-252-3400 ext. 4648

Fax: 514-252-3569

E-mail: [gbernier.hmr@ssss.gouv.qc.ca](mailto:gbernier.hmr@ssss.gouv.qc.ca)

**SUPPLEMENTARY METHODS**

**Fixation, sectioning, and immunolabeling**

For fixation, tissues were immersed for 1 h at room temperature in 4% paraformaldehyde (PFA)/3% sucrose in 0.1 M phosphate buffer, pH 7.4. Samples were washed three times in PBS, cryoprotected in PBS/30% sucrose, and frozen in CRYOMATRIX embedding medium (CEM) (Thermo Shandon, Pittsburgh, PA). Otherwise, tissues were fixed in 10% buffered formalin and embedded in paraffin according to standard protocols. 5 to 7 µm thick sections were mounted on Super-Frost glass slides (Fisher Scientific) and processed for immunofluorescence or immunohistochemistry staining. For immunofluorescence labeling, sections were incubated overnight with primary antibody solutions at 4ºC in a humidified chamber. After three washes in PBS, sections were incubated with secondary antibodies for 1 h at room temperature. Slides were mounted on coverslips in DAPI-containing mounting medium (Vector Laboratories, CA). For immunohistochemistry labeling, formalin-fixed paraffin-embedded slices were analyzed by using the Vectastain® ABC kit (Vector) according to the manufacturer instructions. Peroxidase substrates used are the Vector® VIP (Pink) (Vector), and DAB (brown) (Sigma). Observations were made under a fluorescence microscope (Leica DMRE, Leica Microsystems) and images were captured with a digital camera (Retiga EX; QIMAGING; with OpenLab, ver.3.1.1 software; Open-Lab, Canada). Antibodies used in this study were mouse anti-NeuN, anti-8-oxo-guanine and mouse anti-p16Ink4a (Chemicon), rabbit anti-GFAP (DAKO), rabbit anti-cleaved caspase-3 (Cell Signaling), and mouse anti-mouse Bmi1 (Abcam). Secondary antibodies used were FITC-conjugated donkey anti-mouse and rhodamine-conjugated donkey anti-rabbit (Chemicon).

**RT-PCR and quantitative real-time PCR**

All primers were designed to flank individual exons and tested by PCR in RT+ and RT− control extracts. Total RNA was isolated using TRIzol reagent (Invitrogen). Reverse transcription (RT) was performed using 1 µg of total RNA and the MML-V reverse transcriptase (Invitrogen). Real-time PCR was performed using the Platinum SYBRGreen SuperMix (Invitrogen) and a Real-Time PCR apparatus (BioRad). Primers sets used were as follow:

| Genes | Forward | Reverse |
| --- | --- | --- |
| Bmi1 | GGAGACCAGCAAGTATTGTCCTATTTG | CATTGCTGCTGGGCATCGTAAG |
| p16 | CAACGCCCCGAACTCTTTC | GCAGAAGAGCTGCTACGTGAAC |
| p19 | GGCTAGAGAGGATCTTGAGAAGAGG | GCCCATCATCATCACCTGGTCCAGG |
| Noxa | AGTTCGCAGCTCAACTCAGG | GCCGTAAATTCACTTTGTCTCC |
| xCT | TGGAGGTCTTTGGTCCTTTG | CCAGGATGTAGCGTCCAAAT |
| GST-1α | CGCCACCAAATATGACCTCT | CCATGGCTCTTCAACACCTT |
| NQO1 | TTCTCTGGCCGATTCAGAGT | GAGTGTGGCCAATGCTGTAA |
| Sod1 | CGGATGAAGAGAGGCATGTT | CACCTTTGCCCAAGTCATCT |
| Sod2 | GGCCAAGGGAGATGTTACAA | GCTTGATAGCCTCCAGCAAC |
| Sestrin1 | TCGTGTGCACTCCTGAAAAG | TACCGGGTAATGGCTCTCAG |
| Sestrin2 | CCTCCTTTGTGTTGTGCTGT | ACGGTTCTCCATTTCCTCCT |
| p21 | TGTCTTGCACTCTGGTGTCTG | ATCTGCGCTTGGAGTGATAGA |
| IL6 | AGTTGCCTTCTTGGGACTGA | TCCACGATTTCCCAGAGAAC |
| hprt | ACTGTAATGATCAGTCAACGGG | GGCCTGTATCCAACACTTGG |

**Western blotting.**

Total protein extracts were prepared in the CompleteMini protease inhibitor cocktail solution (Roche Diagnostics). Proteins contents were quantified using the Bradford reagent. Proteins were resolved in Laemmli buffer by SDS-PAG electrophoresis and transferred to a Nitrocellulose Blotting Membrane (Pall, FL) that was exposed to the primary antibodies. The antibodies used were mouse anti-Bmi1 (Millipore), and mouse anti-β actin (Sigma). Membranes were treated with corresponding horseradish peroxidase-conjugated secondary antibodies (Sigma) and developed using the Immobilon Western (Millipore).

**Determination of reactive oxygen species and lipid peroxidation levels**

Cortices were dissected on ice and cultured embryonic neurons were collected following trypsin treatment (TrypLE^TM^ Express, Invitrogen). Samples were homogenized in a Potter-Elvehjem homogenizer in 5 mM Tris-HCl buffer, pH 7.4 (Merck, Darmstadt, Germany). The dichlorodihydrofluorescein diacetate (DCF-DA; Sigma) staining method was used for the quantification of ROS generation. The dye was added to the homogenates to achieve a final concentration of 25 μM. The change in fluorescence intensity was monitored by using a fluorescence multi-well plate reader (Perseptive Biosystems), at excitation 485 nm and emission 530 nm. On the other hand, homogenates were tested for lipid peroxidation by measuring the concentration of malondialdehyde (MDA) according to the thiobarbituric acid reactive substrate (TBARS) method. The homogenates to 0.6% TBA mixture was boiled for 30 min. After cooling on ice, the chromogenic reaction was read in a spectrophotometer (Beckman) at 535 nm. Data are normalized to the amounts of proteins per sample, and are expressed as fold change relative to young or WT values, respectively. Results are Mean ± s.d..

**Chromatine Immunoprecipitation (ChIP) analyses**

Primers used for the CHIP assay were: x-CT (-193 to + 15); (F) 5'-ATCCATTGAGCAACCCACA -3' and (R) 5'-AGCTGAGCTGGTGTGTAATG-3', and Sod2 (-537 to + 23); (F1) 5'-CAAACCTGCGACGTGATTTA-3', (R1) 5'-AGCTGCAAAGCTTCCACTCT-3', (F2) 5'-AATTTGGCACAGGGGAGAC-3' and (R2) 5'-CGCCCGACACAACATTATT-3' for site 1 and site 2, respectively, and β-major (F) 5'-CAGTGAGTGGCACAGCATCC-3', (R) 5'-CAGTCAGGTGCACCATGATGT-3'.
